# Reply to E.C. Dee et al

**DOI:** 10.1200/GO.23.00102

**Published:** 2023-06-08

**Authors:** Bogda Koczwara, Alexandre Chan, Michael Jefford, Wendy W.T. Lam, Carolyn Taylor, Claire E. Wakefield, Nirmala Bhoo Pathy, Bishal Gyawali, Gregory Harvet, Yan Lou, C.S. Pramesh, Miyako Takahashi, Ke Yu, Raymond J. Chan

**Affiliations:** Bogda Koczwara, MBioethics, Department of Medical Oncology, Flinders Medical Centre, College of Medicine and Public Health, Flinders University, Adelaide, Australia; Alexandre Chan, PharmD, MPH, School of Pharmacy and Pharmaceutical Sciences, University of California, Irvine, CA, Department of Oncology Pharmacy, National Cancer Centre Singapore, Singapore; Michael Jefford, MBBS, PhD, MPH, MHlthServMt, Department of Health Services Research and Australian Cancer Survivorship Centre, Peter MacCallum Cancer Centre, Melbourne, Victoria, Australia, Sir Peter MacCallum Department of Oncology, University of Melbourne, Melbourne, Victoria, Australia; Wendy W.T. Lam, MSc, Jockey Club Institute of Cancer Care, LKS Faculty of Medicine and Centre for Psycho-oncology Research and Training, School of Public Health, The University of Hong Kong, Hong Kong, China; Carolyn Taylor, BFA, Global Focus on Cancer, South Salem, NY; Claire E. Wakefield, PhD, MPH, School of Clinical Medicine, UNSW Medicine & Health, Discipline of Paediatrics and Child Health, Sydney, Australia, Behavioural Sciences Unit, Kids Cancer Centre, Sydney Children’s Hospital, Randwick, Australia; Nirmala Bhoo Pathy, MD, PhD, Centre for Epidemiology and Evidence Based Practice, Faculty of Medicine, University of Malaya, Kuala Lumpur, Malaysia; Bishal Gyawali, MD, PhD, Queen’s Global Oncology Program, Department of Oncology, Queen’s University, Kingston, Canada; Gregory Harvet, MD, Department of Paediatrics, Centre Hospitalier Territorial, Noumea, New Caledonia; Yan Lou, PhD, RN, School of Nursing, Department of Medicine, Hangzhou Normal University, Hangzhou, Zhejiang Province, China; C.S. Pramesh MS, Tata Memorial Centre, Homi Bhabha National Institute, Mumbai, India; Miyako Takahashi, MD, PhD, Japan Cancer Survivorship Network, Tokyo, Japan, Iwate Medical University, Iwate, Japan; Ke Yu, PhD, Tokyo Jikei University School of Medicine, Tokyo, Japan; and Raymond J. Chan, PhD, RN, Caring Futures Institute, College of Nursing and Health Sciences, Flinders University, Bedford Park, Australia

We thank Dee et al^1^ for their comments relating to our paper.

We agree that political determinants (as well as commercial and social determinants) are important considerations for advancing survivorship care in the region. Indeed, due considerations of political determinants are not unique to the Indo-pacific region nor to survivorship care alone.^2^ Across many health contexts, future successes will likely require coordinated, top-down and bottom-up approaches involving local, regional, and global collaborations.

Dee and colleagues point out that, similarly to cancer care elsewhere, technology offers many opportunities for the advancement of health care, well beyond survivorship care, and we particularly appreciate their reference to frugal innovation given the ubiquitous resource limitations. As they emphasize, technological innovation is often enabled by infrastructure investment which in turn is dependent on the local political (and commercial) context. We agree that innovations in technology could help lower-resourced countries leapfrog the relatively slower progress made by other countries in the past.

We thank the authors for emphasizing that survivorship care (and cancer care in general) does not occur in a vacuum but should be viewed within a complex geosociopolitical context. Any advancements require adaptation to the local context and consideration of factors proximal and distal to acute cancer care delivery. These considerations are useful when considering how to improve survivorship care not just in the Indo-Pacific region but throughout the globe.

## References

[1] Dee EC Ho FDV Yee K et al Survivorship care for people with cancer in the Indo-Pacific: The imperative to harness political determinants, international exchange, and technological innovation JCO Glob Oncol 10.1200/GO.23.00052 2023 10.1200/GO.23.00052 PMC10497291 37290023

[2] Kickbusch I The political determinants of health—10 years on BMJ 350 h81 2015 25569203 10.1136/bmj.h81

